# Mast cell tryptase induces nuclear remodelling and reduced growth in breast cancer cells

**DOI:** 10.1038/s41420-025-02813-1

**Published:** 2025-10-27

**Authors:** Filip Pano, Laura Bub, Débora Parrine, Aida Paivandy, Anna-Karin Olsson, Gunnar Pejler, Fabio Rabelo Melo

**Affiliations:** 1 Uppsala University, Department of Medical Biochemistry and Microbiology, Uppsala, Sweden; 2 Uppsala University, Department of Organismal Biology, Uppsala, Sweden; 3 Uppsala University, Department of Medical Sciences, Uppsala, Sweden

**Keywords:** Breast cancer, Granulocytes

## Abstract

Mast cells accumulate in breast cancer, but there is only limited knowledge of how they impact on breast cancer growth. Here we show that tryptase, a major compound stored in mast cell secretory granules, has profound effects on breast cancer cell morphology and growth, the latter by a combination of anti-proliferative and pro-apoptotic effects. Mechanistically, we show that tryptase is taken up by breast cancer cells, and enters their nuclei. Further, tryptase was shown to cause major effects on chromatin organization, and to induce truncation of core histone-3 (H3). H3 truncation was accompanied by reduced levels of epigenetic marks associated with H3. In vivo, tryptase-positive mast cells were found in PyMT breast cancer tumours and in human triple negative breast cancer, and a proliferation clearance zone was seen in the vicinity of tryptase-positive mast cells. It was also observed that mast cells were activated to a higher extent in breast cancer tumours than in healthy tissue. Finally, ATAC-seq analysis revealed that tryptase affected chromatin accessibility at regions of the genome associated with genes known to influence breast cancer growth. Altogether, the present study introduces a mechanism for how mast cell tryptase can regulate breast cancer cell growth.

## Introduction

Mast cells (MCs) are versatile effector cells of the immune system. They are implicated in the host defence against bacteria, parasites and various types of toxins, but are particularly well-known for their detrimental impact during allergic conditions, including asthma [1–3]. In addition to their aggravating effects in allergic settings, MCs have also been demonstrated to contribute in various other types of pathologies, including atherosclerosis, contact dermatitis, arthritis, diabetes and psoriasis [4–9]. Moreover, there is a large documentation showing that MCs frequently accumulate in various types of malignancies, including melanoma, prostate cancer, lung cancer, Hodgkin’s lymphoma and also in breast cancer (reviewed in refs. [10–14]).

Although the presence of MCs in tumours has been recognized for over a century, there is still relatively limited knowledge of how MCs may influence tumour growth. To address the latter issue, the presence of MCs in tumours has been correlated with tumour prognosis. In many such cases, MC presence has been associated with worsened outcome, indicating a detrimental impact of MCs in the respective conditions [10–14]. However, there are also numerous studies indicating the opposite, i.e., that MC presence can be linked to an improved prognosis, i.e., that MCs in fact may have protective effects against tumour progression [10–14].

In breast cancer, many studies performed to date suggest that MCs have a protective role. For example, MCs were connected with a favourable prognosis in a study based on over 4000 cases of invasive breast cancer [15] and there are several additional studies in which MCs have been linked to a better disease outcome [16–23]. However, although the collected evidence from these clinical studies supports a protective effect of MCs against breast cancer, the mechanism(s) by which MCs may suppress breast cancer growth is not known in detail. It should also be noted that the role of MCs in breast cancer may be highly complex, as manifested by a number of studies suggesting a detrimental impact of MCs, for example, by mediating resistance to chemotherapy and as promoters of angiogenesis/lymphangiogenesis (reviewed in ref. [14]).

A hallmark feature of MCs is their high content of secretory granules, which contain large amounts of a panel of preformed compounds, including various biogenic amines (histamine, serotonin, dopamine), certain preformed cytokines/growth factors, lysosomal enzymes, proteoglycans and a number of MC-restricted proteases. The MC-restricted proteases include tryptase, chymase and carboxypeptidase A3, which are all stored at remarkably high levels in the granules [24, 25]. Hence, when MCs are activated by mechanisms causing MC degranulation, large amounts of these proteases are released to the exterior, and it is thus likely that they may have an extensive impact on the surrounding tissue, most likely through proteolytic effects. Previous studies focusing on the relation between tryptase and breast cancer have indicated an association between serum tryptase levels and extent of tumour angiogenesis [26]. Further, the density of tryptase-positive mast cells in tumour tissues has been linked to the extent of tumour angiogenesis in breast cancer [27, 28]. However, the potential functional impact of tryptase on breast cancer has not been extensively studied.

In previous studies, we have shown that MC tryptase can regulate cell proliferation, both intrinsically in MCs and also by affecting other cells [29–32]. Based on these observations, we here hypothesized that tryptase might have the capacity to regulate the growth of breast cancer cells. Indeed, our findings reveal that tryptase has anti-proliferative and pro-apoptotic effects on breast cancer cells, and that this is associated with uptake of tryptase into the tumour cell nuclei.

## Results

### MC tryptase affects breast cancer cell morphology

**Fig. 1:**
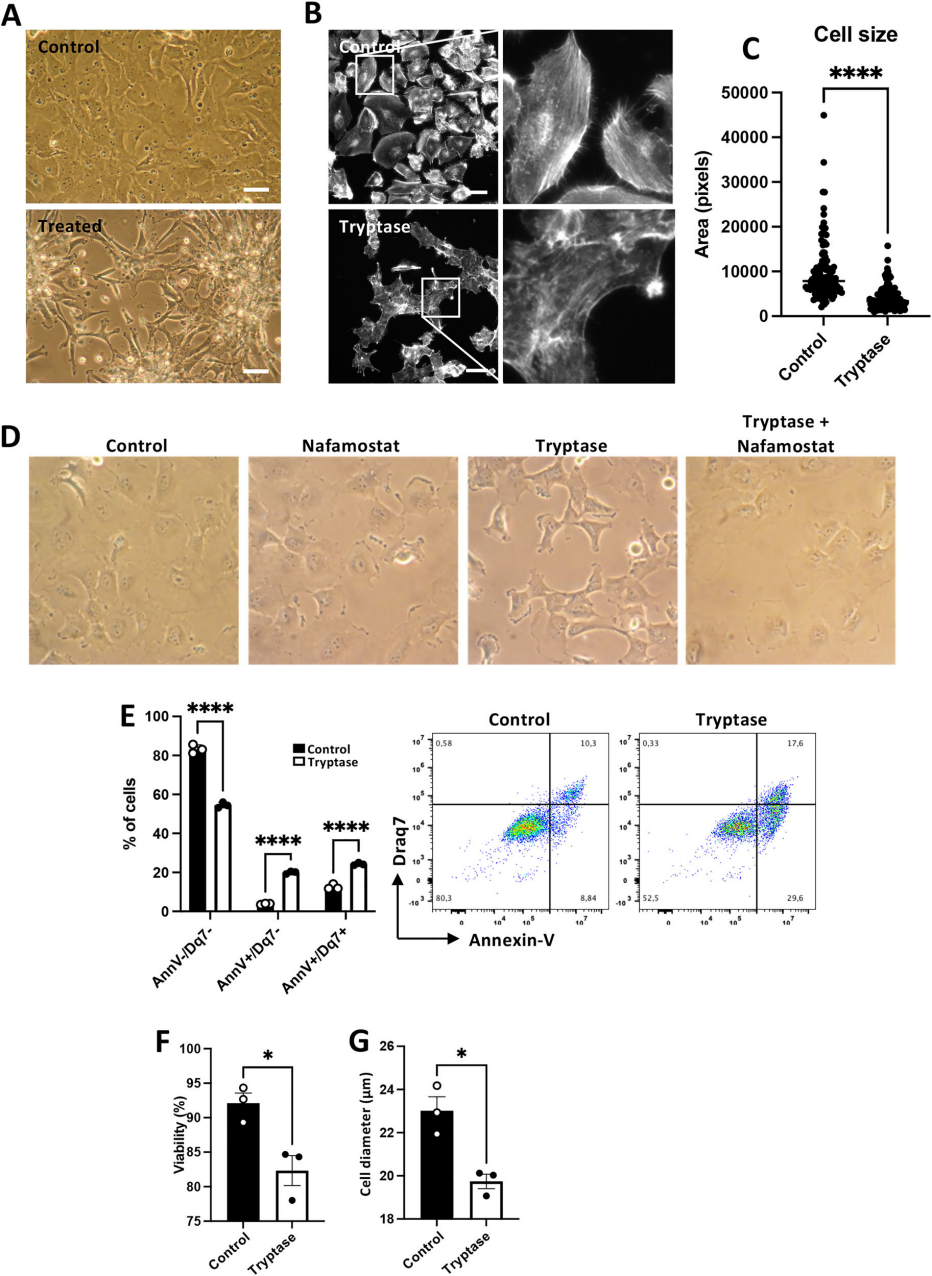
MC tryptase affects breast cancer cell morphology and induces cell death in breast cancer cells. Hs578T breast cancer cells were cultured for 48 h under normal conditions (Control) or with 50 nM tryptase. **A** Morphological changes in Hs578T cells following tryptase treatment. Scale bar: 25 μm. **B** F-actin staining of Hs578T cells, showing cytoskeletal changes after treatment with tryptase. Scale bar: 25 μm. **C** Cell size was determined by quantifying the area of each cell in pixels. **D** Nafamostat prevents tryptase-induced morphological changes in Hs578T cells. **E** Cell viability analysis using Annexin V (AnnV) and Draq7 (Dq7) staining. Viable cells (AnnV^-^/Dq7^-^), apoptotic cells (AnnV^+^/Dq7^-^) and late apoptotic/necrotic cells (AnnV^+^/Dq7^+^) **F** Cell viability determined by trypan blue exclusion. **G** Cell size measurement (diameter; μm). Data are expressed as mean values ± SEM (*n* = 3) and represent at least three independent experiments. Statistical significance was determined by unpaired *t*-test; *p ≤ 0.05; ****p ≤ 0.0001.

To assess the possible impact of tryptase on breast cancer cells, we first investigated if exogenously added human MC tryptase may have an effect on basic morphological features of Hs578T breast cancer cells. As seen in Fig. 1A, addition of tryptase to the breast cancer cells caused profound effects on cellular morphology, as manifested by a marked contraction of the tumour cells, accompanied extensive cell clumping. Such effects were notable starting from ~24 h after tryptase addition to the cell cultures and persisted at least up to 72 h, with maximal effects seen at ~48 h (Fig. 1A). To obtain further insight into this effect, cells were stained for F-actin. As depicted in Fig. 1B, untreated cells displayed extensive filamentous actin structures, and F-actin-positive contacts between adjacent cells were abundant. However, after treatment with tryptase, the filamentous appearance of the F-actin staining was essentially abrogated, and the F-actin-positive intercellular contacts were abolished. In line with the effect on cellular morphology, image analysis followed by quantification demonstrated a profound and statistically significant reduction in cellular size after treatment of the breast cancer cells with tryptase (Fig. 1C). To assess whether these observed effects were dependent on the enzymatic activity of tryptase, the impact of a selective tryptase inhibitor (nafamostat) was investigated. These experiments revealed that the effects of tryptase on Hs578T cell morphology were completely abrogated in the presence of the tryptase inhibitor (Fig. 1D), indicating that the effects of tryptase on breast cancer cell morphology are dependent on its enzymatic activity.

### MC tryptase induces cell death in breast cancer cells

To further assess the impact of tryptase on the breast cancer cells, we investigated whether tryptase can affect their viability. Indeed, the addition of tryptase to the tumour cells resulted in a reduction in the proportion of viable cells, accompanied by the appearance of cells undergoing apoptosis-like cell death (Annexin V^+^/Draq7^-^). In addition, tryptase treatment caused an expansion of cell populations double positive for Annexin V and Draq7, the latter indicative of necrosis/late apoptosis (Fig. 1E). Decreased cell viability after tryptase treatment was also confirmed by Trypan Blue exclusion (Fig. 1F). In accordance with the effects noted by light microscopy analysis (see Fig. 1A, B), tryptase was also found to cause a marked reduction of cell size (diameter) (Fig. 1G).

### MC tryptase has anti-proliferative effects on breast cancer cells

**Fig. 2:**
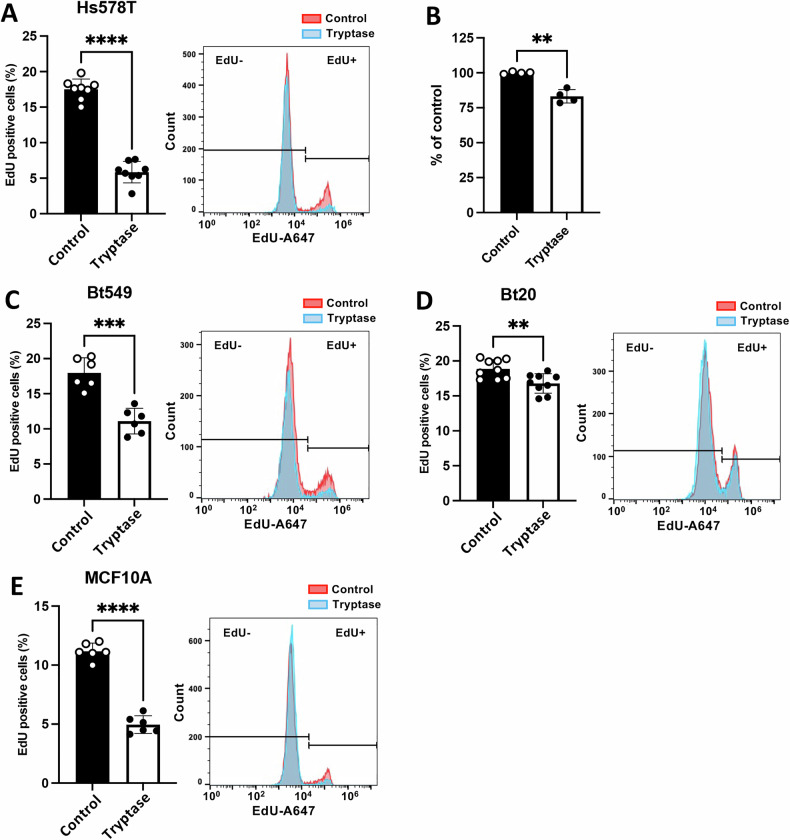
MC tryptase has anti-proliferative effects on breast cancer cells. Cell proliferation was assessed by EdU incorporation in both breast cancer and in a non-transformed cell line of mammary origin, after 48 h of culture under normal conditions (Control) or with 50 nM tryptase. Triple-negative breast cancer cells: **A** Hs578T, **C** Bt549 and **D** Bt20. **E** Non-tumorigenic breast epithelial cell line: MCF10A. **B** Hs578T cell viability measured using a resazurin-based reagent (PrestoBlue). Data represent at least three independent experiments and are given as mean values ± SEM (*n* = 6-9). Statistical significance was determined by unpaired *t*-test; **p ≤ 0.01; ***p ≤ 0.005; ****p ≤ 0.0001.

Next, we asked whether tryptase, in addition to inducing breast cancer cell death, also can have anti-proliferative effects on such cells. Proliferation was assessed by the EdU assay, which is based on measurements of DNA synthesis by incorporation of a thymidine nucleoside analogue, followed by a fluorophore-labelled reaction to allow detection by flow cytometry. As seen in Fig. 2A, tryptase caused a substantial reduction in the proliferation of Hs578T cells. As assessed by the PrestoBlue assay, tryptase also caused a marked decrease in the metabolic activity of cells, in support of an anti-proliferative effect of tryptase on the breast cancer cells (Fig. 2B). In addition, a significant reduction of proliferation was seen in tryptase-treated Bt549, Bt20 breast cancer cells and in a non-tumourigenic cell line of mammary origin MCF10A (Fig. 2C-E). Hence, MC tryptase has the ability to suppress the proliferation of multiple types of breast cancer cells.

### Tryptase is taken up by breast cancer cells and can enter the tumour cell nucleus

**Fig. 3:**
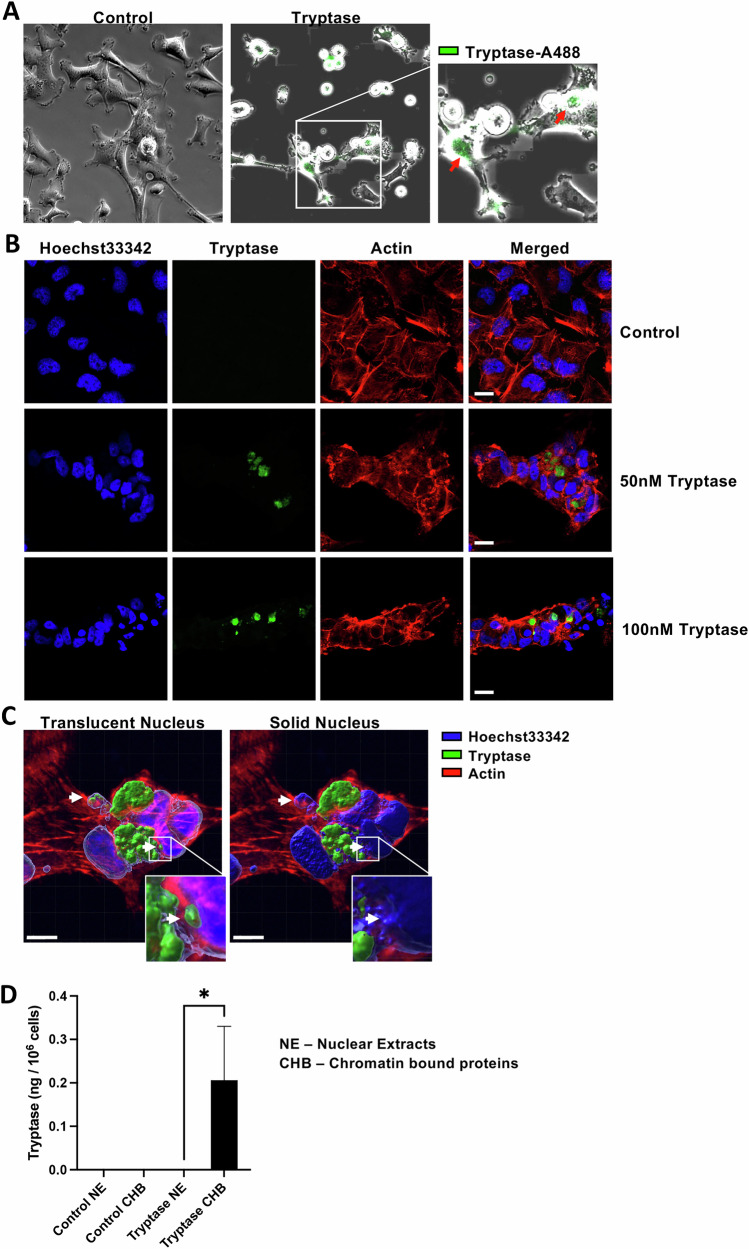
Tryptase is taken up by breast cancer cells and can enter the tumour cell nucleus. Hs578T cells were cultured for 48 h under normal conditions (Control) or with 50 nM tryptase. **A** Image of Hs578T cells treated with Alexa-488-labelled tryptase. Red arrows indicate tryptase binding to the cells. **B** Immunofluorescence staining of tryptase in Hs578T cells; Hoechst 33342 staining was applied to visualize the nucleus and actin staining was used to visualize the cytoskeleton. **C** Confocal Z-stack images used to generate 3D images of tryptase-treated Hs578T cells. Two modes are shown: translucent nucleus (left) and solid block (right). In the solid block image, nuclear staining for tryptase (white arrow) is concealed, confirming its intra-nuclear localization. **D** Tryptase levels were quantified by ELISA using extracts from cell fractionation. Data represent two independent experiments and are given as mean values ± SEM (*n* = 4). Statistical significance was determined by unpaired *t*-test; *p ≤ 0.05.

To approach the mechanism by which tryptase affects breast cancer cells, we investigated the interaction between tryptase and tumour cells. To this end, tryptase was fluorescently labelled and added to the cells. By using time lapse microscopy, we noted that tryptase bound to the breast cancer cells in a time-dependent manner (Fig. 3A and Supplementary Videos 1 and 2). Moreover, we noted that tryptase was in fact taken up by the tumour cells (Fig. 3A and Supplementary Videos 1 and 2). The latter finding was somewhat unexpected, being in contrast to a more expected scenario in which tryptase may affect target cells by interacting with cell surface compounds such as protease-activated receptors [33–35].

To obtain deeper insight behind the latter observation, we stained tryptase-treated cells with an anti-tryptase antibody, and used confocal microscopy to visualize the localization of tryptase. This approach confirmed that tryptase indeed is taken up by the tumour cells, and also confirmed profound effects of tryptase on the tumour cell morphology (Fig. 3B). Even more strikingly, it was observed that tryptase could be found in the cancer cell nuclei (Fig. 3C). The presence of tryptase in the nucleus of the cells was also supported by subcellular fractionation of the cells, followed by quantification of tryptase in the nuclear compartment of non- and tryptase-treated cells by employing ELISA (Fig. 3D). To further substantiate the presence of tryptase within the nuclear compartment, tryptase was visualized in cells where the nucleus was depicted either in a translucent or solid block mode, with the solid depiction being a mode in which the surface of the nucleus is concealed by the software. Hence, if the antibody is bound to tryptase present on the surface of the nucleus, it will be visible in the solid block mode, whereas if the antibody binds to tryptase located within the interior of the nucleus, image conversion to the solid block mode will conceal the staining. As seen in Fig. 3C, the solid mode of visualization led to abrogated tryptase staining, supporting that tryptase is in fact found within the interior of the nucleus, rather than on the nuclear surface.

### Tryptase affects chromatin organization in breast cancer cells

**Fig. 4:**
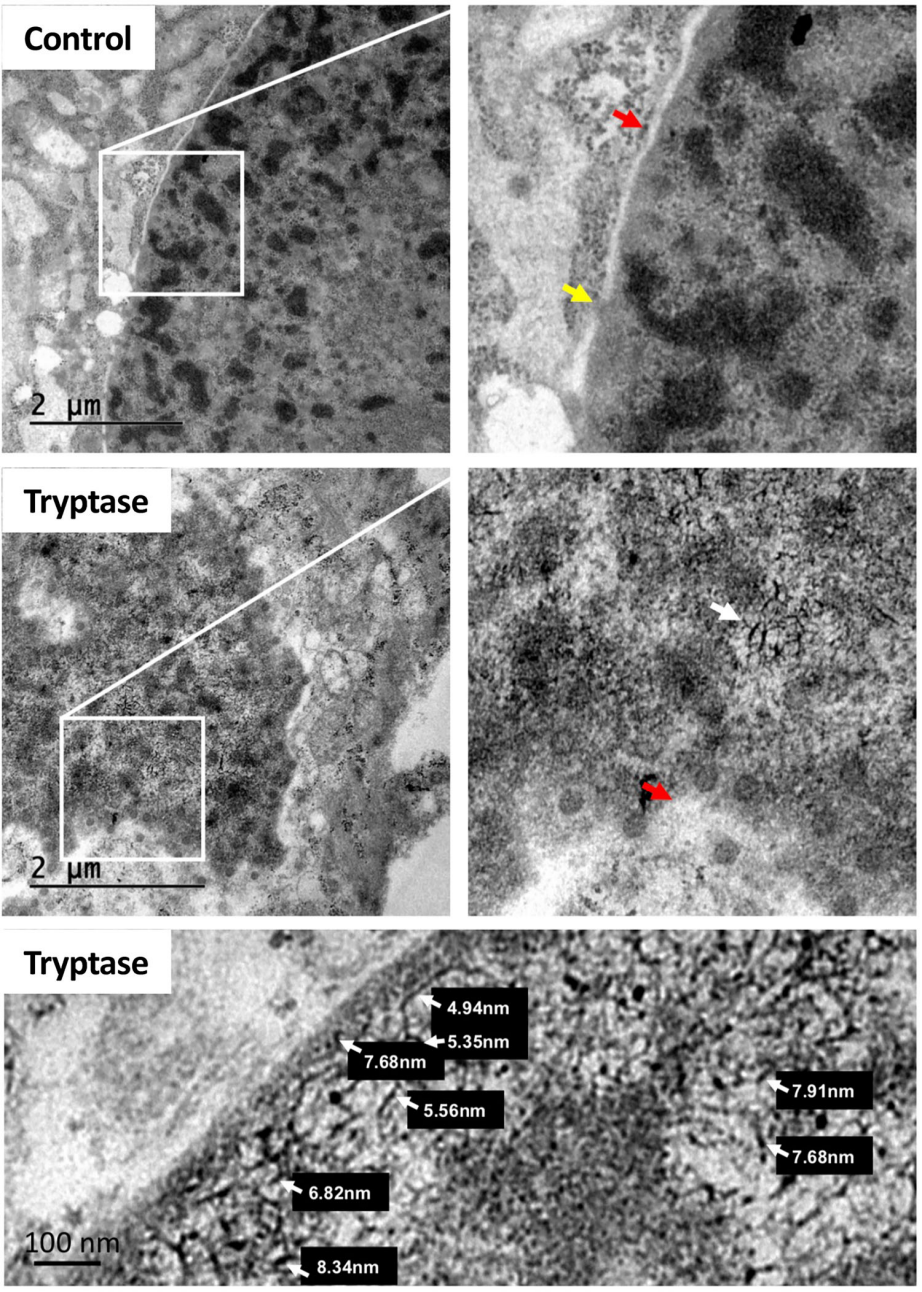
Tryptase affects chromatin organization in breast cancer cells. Hs578T cells were cultured for 48 h under normal conditions (Control) or with 50 nM tryptase. Transmission electron microscopy (TEM) images of control- and tryptase-treated cells. In control cells, the nuclear envelope is intact (red arrow) and nuclear pores are visible (yellow arrow). In tryptase-treated cells, the nuclear envelope appears less defined (red arrow) with fewer nuclear pores and chromatin exhibiting a fibrillar-like structure (white arrow). The lower panel shows quantifications of the diameters of fibrillar-like chromatin, compatible with a beads-on-a-string organization.

To provide more detailed insight into the effects of tryptase on breast cancer cells, in particular to assess for potential effects on the tumour cell nuclei, we used transmission electron microscopy (TEM). These analyses showed that non-treated breast cancer cells displayed normal nuclear structure, with an intact nuclear envelope, visible nuclear pores and clear distinction between areas dominated by euchromatin and heterochromatin, respectively (Fig. 4). In contrast, the nuclei of tryptase-treated tumour cells showed an aberrant morphology, with less clear distinction between euchromatin and heterochromatin, and also with a less defined nuclear envelope and less abundance of nuclear pores. Moreover, the chromatin in tryptase-treated cells had a more fibrillar-like appearance than in controls, compatible with conversion of the chromatin into a beads-on-a-string organization, i.e., with separated nucleosomes. To substantiate this possibility, the diameters of the fibrillar-like structures were measured and, indeed, their diameters were similar to the known diameter of individual nucleosomes (~5-8 nm). Altogether, tryptase has thus the capacity to cause major nuclear remodelling in breast cancer cells.

### Tryptase-positive MCs are present in PyMT breast cancer tumours

**Fig. 5:**
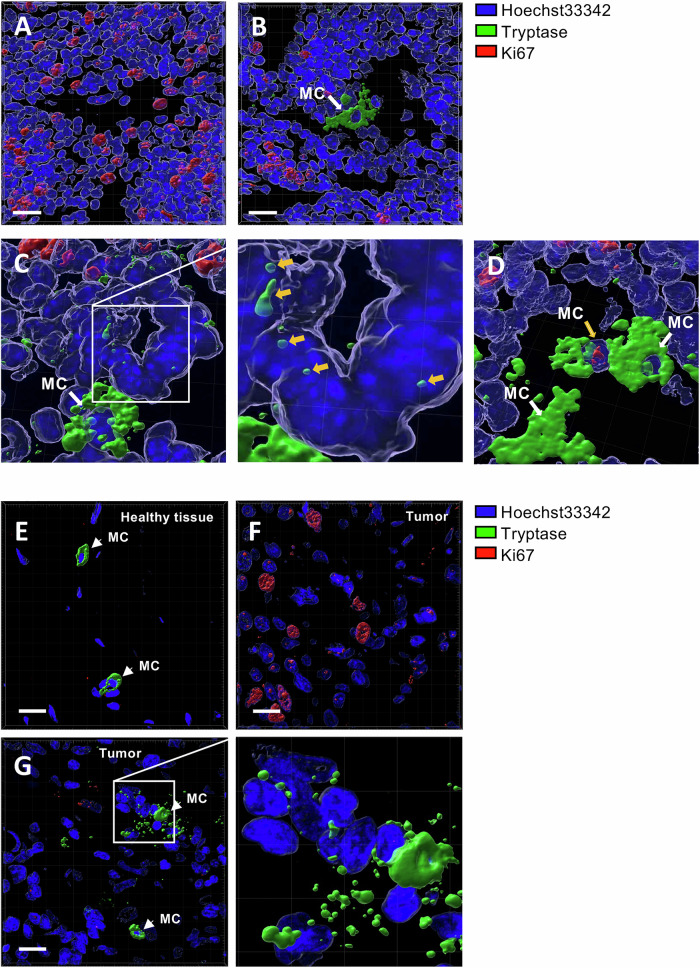
Tryptase-positive MCs are present in PyMT murine breast cancer tumours and human triple-negative breast cancer. Confocal Z-stack images stained with Hoechst 33342 (blue), tryptase (green) and Ki67 (red) were used to generate 3D-images of PyMT murine breast cancer tumour sections (**A**-**D**), healthy mammary tissue (**E**) and human triple-negative breast cancer tumours (**F**, **G**). **A** PyMT tumour tissue lacking MCs. **B** Reduced Ki67 positivity near tryptase-positive MCs. **C** Nuclear localization of tryptase within tumour cells (yellow arrows). **D** Ki67-positive MCs detected within the tumour (yellow arrow). **E** Healthy human breast tissue. **F** Human breast cancer tumour section lacking MCs. **G** Tumour tissue with reduced Ki67 positivity near tryptase-positive MCs.

The data above suggest that tryptase can have an impact on breast cancer cells. To provide in vivo insight into this, we assessed whether tryptase-positive MCs can be found within breast cancer tissue recovered from the PyMT mouse model, a model in which breast cancer develops spontaneously [36]. As seen in Fig. 5A-D, tryptase (Mcpt6)-positive MCs were indeed found within the tumour tissue. Further, it was observed that tryptase was present not only within MCs, but also in the surrounding tissue (Fig. 5C), indicating release of tryptase from the tumour-associated MCs. Moreover, it was observed that tryptase could in fact be found within the interior of tumour cell nuclei (Fig. 5C), i.e., in agreement with the results above showing that exogenously added tryptase can be taken up by breast cancer cells and enter their nuclei (see Fig. 3C). The nuclear localization of tryptase within tumour cells was confirmed by using the solid block/translucent approach (see Fig. 3C; not shown). To assess for tumour cell proliferation, we stained the tissue for Ki67. As expected, extensive Ki67 positivity was seen in the tumour tissue (Fig. 5A). However, it was observed that Ki67 positivity was markedly reduced in the vicinity of tryptase-positive MCs (Fig. 5B), in agreement with an anti-proliferative impact of tryptase on the tumour cells in the local environment. It was also notable that Ki67-positive MCs were detected within the tumour (Fig. 5D), indicating that tumour-associated MCs have the capacity to proliferate.

### Tryptase-positive MCs are present in triple-negative breast cancer and are associated with reduced proliferation of tumour cells

To provide further insight into the possible impact of MC tryptase on breast cancer, we also stained human triple-negative breast cancer tissue for tryptase. As controls, mammary tissue from healthy control tissue was stained. As displayed in Fig. 5E-G, tryptase-positive MCs could be found in both the healthy tissue and in the triple-negative breast cancer. The tumour-associated MCs were generally positive also for avidin, a compound known to bind to anionic proteoglycans within MC granules [37] (Supplementary Fig. 2). Moreover, the MCs were frequently double-positive for chymase, a highly MC-specific protease that colocalized with tryptase in certain MC subpopulations [38] (Supplementary Fig. 2). Notably, MCs were generally intact (non-activated) in the healthy tissue (Fig. 5E) whereas extensive MC degranulation (activation) was seen in MCs present in the tumour tissue (Fig. 5G). Similar to the murine PyMT tumours, tryptase was widely distributed in the human tumour tissue, and it was also observed that tryptase could be found within tumour cells, indicating uptake. Moreover, it was observed that tryptase could be found within the interior of the tumour cell nuclei (Fig. 5G), with the nuclear localization of tryptase within tumour cells being confirmed by the solid block/translucent approach (Supplementary Fig. 1). It was notable that abundant Ki67 positivity was seen in MC-poor regions of the tumours (Fig. 5F), whereas markedly less Ki67 staining was seen in the vicinity of activated MCs. Hence, these findings show that activated, tryptase-positive MCs populate triple-negative breast cancer tissue, and our findings suggest that such MC populations may have the capacity to regulate tumour cell proliferation.

### Tryptase can execute H3 clipping and can erase epigenetic marks deposited on H3

**Fig. 6:**
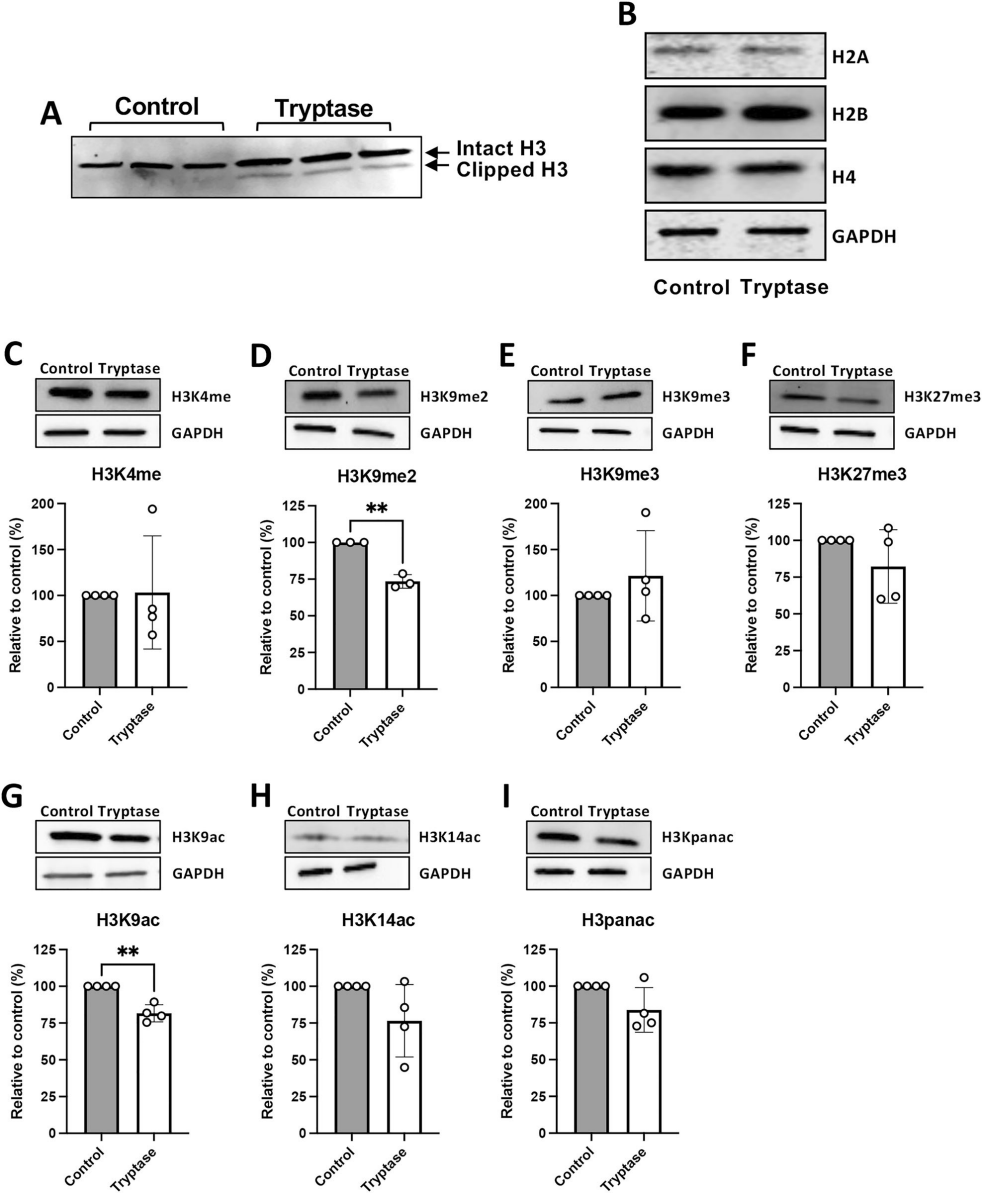
Tryptase can execute H3 truncation (clipping) and erase epigenetic marks on H3. Hs578T breast cancer cells were cultured for 48 h under normal conditions (Control) or with 50 nM tryptase. **A** Western blot analysis of core histone H3, showing the presence of truncated (clipped) H3 in tryptase-treated cells. **B** Western blot analysis of core histones H2A, H2B and H4 in control- and tryptase-treated cells. **C**-**F** Western blot analysis of H3 methylation marks: H3K4me, H3K9me2, H3K9me3 and H3K27me3. **G**–**I** Western blot analysis of major H3 acetylation marks: H3K9ac, H3K14ac and pan-H3K acetylation (H3K4panac). **C**-**I** Densitometric quantification, normalized to GAPDH and expressed as a percentage of the control, is shown in the lower panels. Statistical significance was determined by unpaired *t*-test; **p ≤ 0.01.

To approach the mechanism by which tryptase affects the nuclear organization in breast cancer cells, we considered the possibility that tryptase may execute proteolytic modifications of nuclear proteins. In previous studies we have shown that tryptase can cleave nucleosomal core histones, in particular histone 3 (H3) [30, 31] and we therefore asked whether core histones can be targets for tryptase in a breast cancer cell context. Indeed, treatment of breast cancer cells with tryptase resulted in the generation of truncated (clipped) H3 (Fig. 6A), suggesting an ability of tryptase to cleave this particular core histone. In contrast, neither of the other core histones, i.e., H2A, H2B or H4 were truncated in response to tryptase, indicating that tryptase specifically truncates H3 among the nucleosomal core histones (Fig. 5B).

It is now established that nucleosomal core histones are crucial sites for epigenetic modification, as mediated by the deposition of various epigenetic marks (e.g. acetylation, methylation, phosphorylation) on their N-terminal tails that protrude outside of the nucleosomes [39, 40]. Hence, we next considered the possibility that the truncation of H3 by tryptase can lead to the eradication of corresponding epigenetic marks. For this purpose, we performed Western blot analyses for a panel of epigenetic H3 marks. Comparisons between samples from untreated vs. tryptase-treated cells indicated that tryptase caused a reduction in the levels of several H3 marks, including H3K9me2, H3K27me3, H3K9ac and H3K14ac, and this reached statistical significance for H3K9me2 (Fig. 6D) and H3K9ac (Fig. 6G). In addition, our data indicated a trend of overall reduced H3 acetylation, as judged by analysis using an antibody that detects overall H3K acetylation (H3Kpanac; Fig. 6I). Altogether, these findings indicate that tryptase has the capacity to cause truncation of H3, thereby causing a reduction in corresponding epigenetic marks deposited on this core histone.

### Effect of tryptase on gene expression patterns in breast cancer cells

**Fig. 7:**
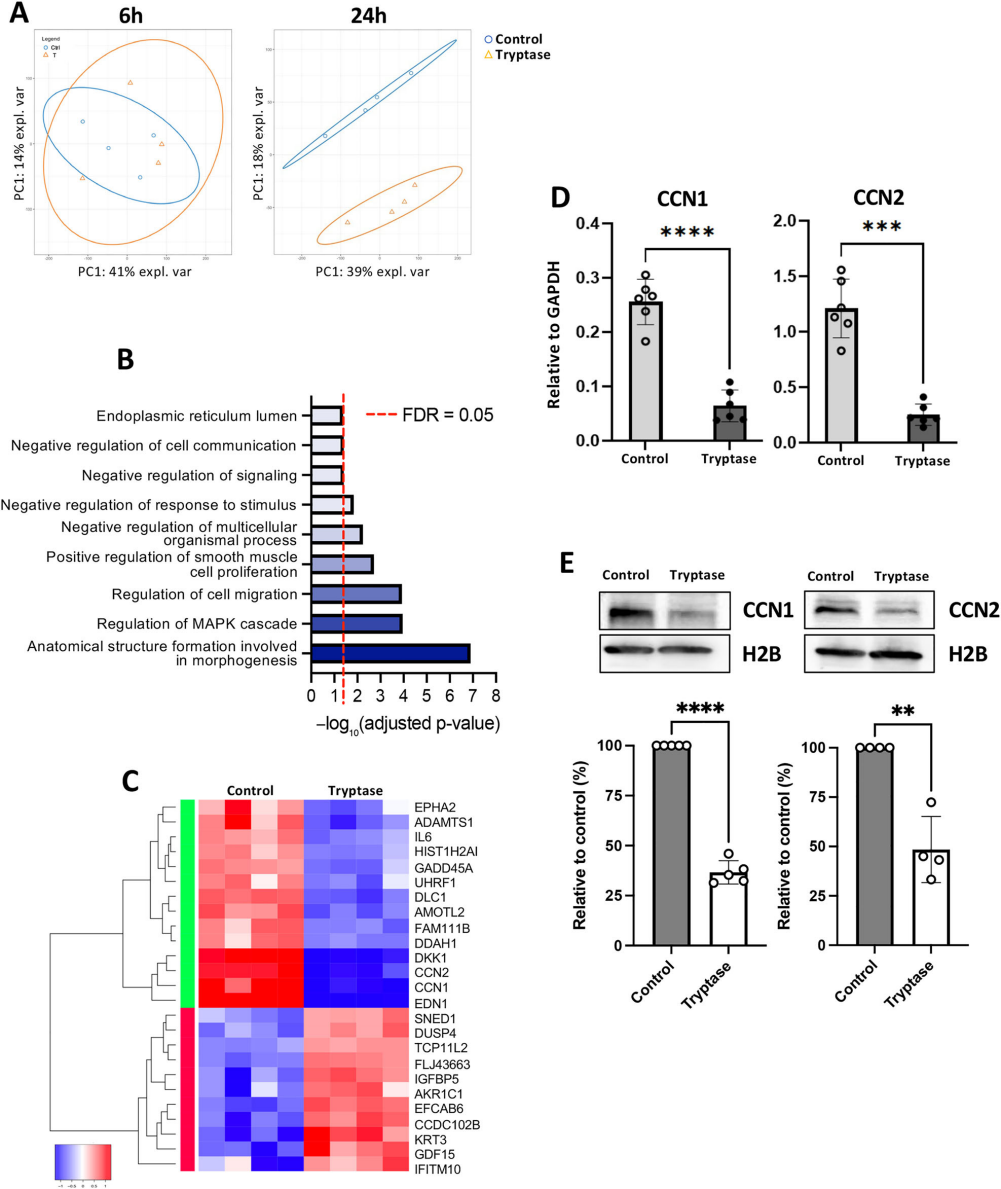
Effect of tryptase on gene expression patterns in breast cancer cells. Transcriptome analysis of Hs578T breast cancer cells cultured for 6 and 24 h under normal conditions (Control) or with 50 nM tryptase. **A** Principal component analysis (PCA) comparing gene expression profiles of control- and tryptase-treated cells. **B** Enriched biological processes in tryptase-treated versus control cells after 24 h treatment. Horizontal bar plot showing the top enriched Gene Ontology (GO) biological processes among the 25 significantly differentially expressed genes (FDR < 0.05). The x-axis represents enrichment significance as -log_10_ (adjusted *p*-value). A red dashed line marks the threshold for statistical significance (adjusted *p* = 0.05). Bar colours represent enrichment strength. **C** Heatmap of transcriptome data, displaying hierarchically clustered genes with 2-log fold changes higher than 2 or lower than -2. Each sample group consisted of four replicates. **D** Quantitative real-time PCR analysis of CCN1 and CCN2 expression levels. **E** Western blot analysis of CCN1 and CCN2, with densitometric quantification normalized to histone H2B and expressed as a percentage of the control, as shown in the lower panels. Statistical significance was determined by unpaired *t*-test; **p ≤ 0.01; ***p ≤ 0.005; ****p ≤ 0.0001.

Next, we considered the possibility that the impact of tryptase on chromatin organization and on the levels of epigenetic marks may be reflected by effects at the level of gene transcription. To assess this, we first performed a genome-wide transcriptome analysis (Ampliseq) of Hs578T breast cancer cells that had been untreated or treated with tryptase for either 6 or 24 h. After 6 h of treatment, no noticeable effects on general gene expression patterns were observed, as visualized by PCA blot analysis (Fig. 7A). In contrast, a robust separation of gene expression patterns between control- and tryptase-treated cells was seen after 24 h (Fig. 7A). Pathway analysis revealed that tryptase predominantly affected pathways related with: Anatomical structure formation involved in morphological characteristics, Regulation of MAPK cascade and Regulation of cell migration (Fig. 7B). Notably, these categories are well in line with our noted morphological and other effects of tryptase on the breast cancer cells.

The transcriptome analysis revealed that altogether 14 genes were significantly downregulated more than 2-fold after tryptase treatment, whereas in total 11 genes showed a significant upregulation (more than 2-fold). Figure 7C depicts a heat map analysis where genes showing the most profound down- or upregulation in response to tryptase are highlighted. As visualized in the analysis, CCN1, CCN2 and EDN1 were among the most robustly downregulated genes, and it is notable that all of these genes have been implicated in breast cancer development [41, 42]. To further substantiate the latter findings, the effect of tryptase on the expression of CCN1 and CCN2 was validated by independent approaches. Indeed, qPCR analysis confirmed a profound downregulation of both CCN1 and CCN2 gene expression after treatment of the cells with tryptase (Fig. 7D). Further, Western blot analysis confirmed the downregulation of CCN1 and CCN2 in tryptase-treated cells at the protein level (Fig. 7E).

### Tryptase affects chromatin accessibility in breast cancer cells

The data above show that tryptase can have marked effects on the nuclear structure in breast cancer cells, and our data also suggest that tryptase can affect gene expression patterns in MCs. One scenario to account for these effects is that tryptase can regulate chromatin accessibility, potentially through proteolytic effects on the core histones (degradation or/and erasing histone marks). To investigate this possibility, we conducted an Assay for transposase-accessible chromatin combined with sequencing (ATAC-seq), a technique that allows the detection and quantification of genomic regions containing open chromatin. ATAC-sec analysis was conducted on samples recovered from Hs578T breast cancer cells that were either non-treated or tryptase-treated (for 48 h).

**Fig. 8:**
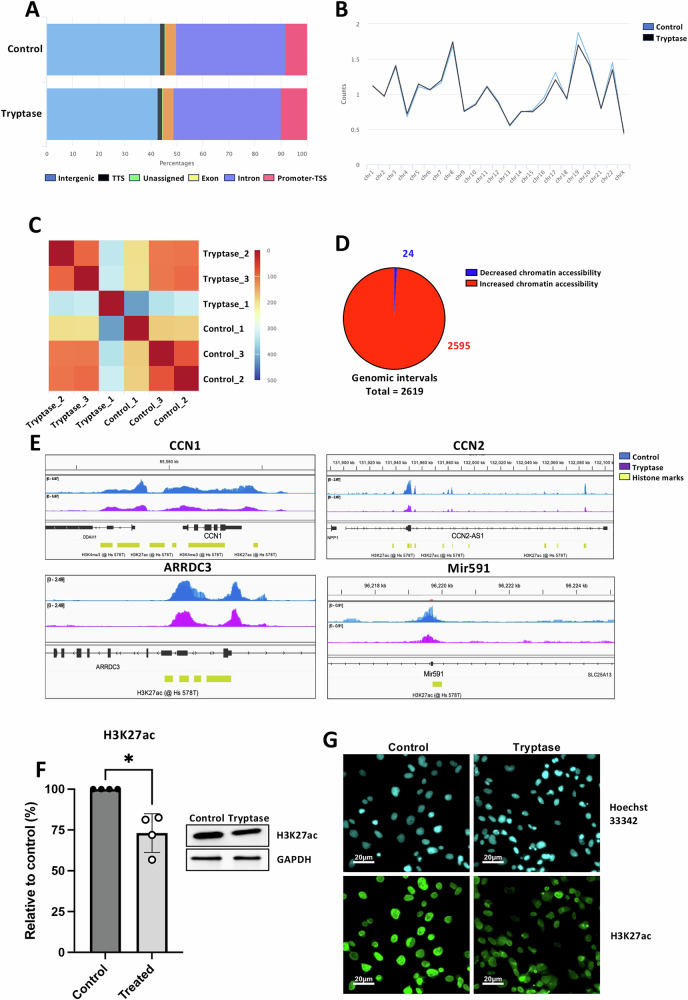
Tryptase affects chromatin accessibility in breast cancer cells. ATAC-seq analysis of Hs578T breast cancer cells cultured for 6 or 24 h under normal conditions (Control 1-3) or with 50 nM tryptase (Tryptase 1-3). **A** Distribution of genomic features assigned to peaks as exon, intergenic, intron, promoter-TSS (transcription starting site), Transcription termination site (TTS) and unassigned. **B** Mapped reads per counts for each chromosome (y-axis, number of mapped reads). **C** Heatmap of the sample-to-sample distances amongst replicates from control and tryptase-treated samples. **D** Number of peaks with decreased (blue) or increased (red) chromatin accessibility. A total of 2,619 genomic intervals were mapped in the analysis. **E** Chromatin accessibility results for control (blue) and tryptase-treated (purple) samples mapped to genes of interest (CCN1, CCN2, ARRDC3, Mir591). A histone mark reference track generated by a compilation of several ChIP-seq studies obtained from ChIP Atlas (www.chip-atlas.org) is displayed in yellow. Peaks are shown as overlayed replicates for each group (control and tryptase). **F** Western blot analysis of H3K27ac, with densitometry quantification normalized to GAPDH and expressed as a percentage of the control. Statistical significance was determined by unpaired *t*-test; *p ≤ 0.05. **G** Immunofluorescence staining for H3K27ac in control and tryptase-treated Hs578T cells after 48 h. Nuclei were visualized with Hoechst 33342 staining. Scale bar: 20 μm.

The distribution of features assignment to sequences was: 43.7% (control) and 42.7% (tryptase) for intergenic sequences, 1.7% in both control- and tryptase samples for transcriptional termination sequences (TTS); 4.2% (control) and 4.3% (tryptase) for exons; 42.1% (control) and 41.2%(tryptase) for introns; 8.2% (control) and 10% for transcription starting sequences (promoter-TSS) (tryptase); 0.1% (control) and 0.2% (tryptase) for unassigned sequences (Fig. 8A). Hence, sequences recovered from control- and tryptase-treated cells presented similar distributions with regard to most features, except for promoter-TSS, for which the sample from tryptase-treated cells had more sequences assigned. The sequences from both datasets were equally distributed amongst the chromosomes, with no significant enrichment in samples from either control- or tryptase-treated cells, except for a slight enrichment in sequences at chromosome 19 in control vs. tryptase-treated cells (Fig. 8B). In Fig. 8C, a heat map is depicted to visualize the replicate similarity when comparing the different samples. This showed that, when comparing the similarities between all samples, the lowest similarity was seen when comparing tryptase-treated-1 with control-1. It was also noted that tryptase-treated replicates-2 and -3 showed high similarity, and that the control samples-1, -2 and -3 showed relatively high similarity (Fig. 8C). To analyse for the overall effect of tryptase on chromatin accessibility over the genome, differential analysis of read numbers for individual sequences was performed by using DESeq2 (Fig. 8D). This revealed that tryptase treatment caused an increased accessibility at altogether 2595 genomic intervals, whereas decreased chromatin accessibility was seen at 24 genomic intervals (Fig. 8D). Hence, tryptase treatment overall results in higher chromatin accessibility across the genome.

We then focused on genes for which the transcriptomic analyses indicated a downregulated expression, as exemplified by CCN1 and CCN2 (see Fig. 7C-E). Indeed, the ATAC-seq analysis revealed higher peaks representing these genes in samples from control compared with tryptase-treated cells (Fig. 8E), indicating that their sequences were less accessible to transcription after treatment of the cells with tryptase. Similar effects of tryptase were also seen for a number of other genes implicated in breast cancer, as shown for ARRDC3 and Mir591 [43, 44] (Fig. 8E).

Since our data above suggest that tryptase has the capacity to regulate the levels of epigenetic histone marks, we asked whether the corresponding peaks in the ATAC-seq analysis associated with the location of specific histone marks. For this, we aligned our data to a histone track for Hs578T available in the repository ChIP-Atlas, containing data from NCBI, DDBJ and ENA (Oki & Ohta, 2015: ChIP-Atlas. https://chip-atlas.org). This analysis revealed that the peaks observed in genes for which tryptase caused a decrease in chromatin accessibility (CCN1, CCN2, ARRDC3, Mir591) all aligned to H3K4me3 and H3K27ac (Fig. 8E). Trimethylation of H3 at lysine 4 (H3K4me3) is a modification which marks the transcription site of active genes, while the acetylation of lysine 27 in histone H3 (H3K27ac) is a marker for active enhancers [45, 46]. To further substantiate these findings, one potential explanation for the selective effects of tryptase on genomic regions associated with H3K27ac could be that tryptase causes truncation of H3 such that H3K27ac marks are erased, in turn leading to chromatin condensation accompanied by effects on transcription of the corresponding genes. To address this possibility, we assessed whether tryptase treatment of the cancer cells can affect the levels of the H3K27ac mark. Indeed, Western blot analysis indicated that tryptase has the capacity to suppress the levels of this epigenetic mark (Fig. 8F), and this notion was also supported by immunohistochemical staining followed by confocal microscopy analysis, revealing a profound decrease in H3K27ac staining after treatment of the breast cancer cells with tryptase (Fig. 8G).

## Discussion

Here we show that MC tryptase has a dampening impact on breast cancer cell growth, as manifested by reduced proliferation and increased rates of cell death. Possibly, tryptase may thus account, at least partly, for the association between MC presence and favourable prognosis in breast cancer. However, it should be emphasized that MCs have the possibility to suppress breast cancer growth through alternative mechanisms, e.g., by secretion of TNF-α and TGF-β [47, 48]. The collective impact of MCs on breast cancer cells (and other types of tumour cells) could thus be a result of the combined action of several mediators released from MCs.

In line with the present study, previous studies have indicated an association between MC presence in breast cancer and reduced tumour cell proliferation [19, 21, 49], and MCs have also been shown to have proapoptotic and cytolytic effects on breast cancer cells [18, 47]. Hence, the data presented here may suggest that tryptase may account for such effects seen in clinical settings. In agreement with this notion, we present in vivo evidence from both a well-established mouse model of breast cancer (PyMT model) and from human triple negative breast cancer, in which tryptase-positive MCs were associated with a distinct proliferation clearance zone within the tumour parenchyma.

It is important to note that MCs have the ability to express and secrete a wide panel of compounds other than tryptase, including various growth factors, arachidonic acid-derived mediators, cytokines and biogenic amines (e.g. histamine), all of which can have the potential to influence breast cancer progression [25, 50]. The collective impact of MCs on breast cancer (or any other type of malignancy) could thus represent combined effects of several MC-expressed compounds. Further, it cannot be excluded that MCs can secrete both anti-tumour and pro-tumourigenic factors, and that the collective impact of MCs in a tumour setting may represent a balance between the action of such factors.

Here we also provide extensive insight into the mechanism by which tryptase negatively impacts on the breast cancer cells. Intriguingly, our data reveal that tryptase in fact is taken up by the tumour cells. This is in contrast to a more expected scenario in which tryptase could potentially act on cell surface receptors, for example, protease-activated receptors such as PAR-2, the latter being a known target for tryptase in multiple contexts [33–35]. Another intriguing finding was that tryptase can enter the nuclei of the breast cancer cells, which was seen both in cultured breast cancer cells as well as in vivo, both in a mouse breast cancer model and in human triple negative breast cancer. Notably, this is in line with our previous findings showing that tryptase can be found intrinsically in the nuclei of MCs [29, 30] and that tryptase can enter the nuclei of melanoma cells [31, 32]. Further, it is demonstrated here that tryptase causes extensive remodelling of the chromatin in cells that have taken up tryptase, as shown by TEM analysis. Intriguingly, it is still not certain how tryptase is taken up and then transported to the nuclear compartment of the breast cancer cells. In a previous study it was shown that tryptase was taken up by endocytosis, as cargo of exosomes, into melanoma cells [31]. We may thus hypothesize that tryptase is taken up into the breast cancer cells through a similar mechanism. However, the mechanism by which tryptase is transported to the nuclear compartment and how it enters the nucleus is still enigmatic. Tryptase does not harbour a canonical nuclear localization signal, and the most likely scenario would thus be that it enters the nucleus as cargo of other compounds. However, the identity of such tryptase-carrying factors remains to be revealed.

Most likely, since tryptase is a proteolytic enzyme, the noted effects of tryptase on chromatin organization may be a result of proteolytic action on nuclear proteins. Indeed, here we demonstrate that tryptase has the capacity to degrade nuclear core histone 3 (H3) within the breast cancer cell nuclei. However, most likely, tryptase entering the nucleus may also have proteolytic actions of additional nuclear targets, but such potential alternative proteolytic targets for tryptase in the nucleus remain to be identified.

Importantly, such proteolytic effects require that tryptase retains enzymatic activity in the intracellular milieu. In support of this, tryptase is known to be resistant to all known endogenous protease inhibitors, due to its tetrameric structure with all of its active sites facing a central, narrow pore [51]. In agreement with the notion that tryptase retains enzymatic activity in the nuclear milieu of the breast cancer cells, we observed core histone 3 (H3) truncation in tryptase-treated breast cancer cells, and it thus appears likely that the effect of tryptase on nuclear organization may be explained by proteolytic modification on nuclear proteins. We also observed that tryptase caused a reduction in the levels of several epigenetic marks deposited on H3. Since epigenetic modification of core histones represents a crucial epigenetic mechanism with large impact on gene expression [39, 40], we considered the possibility that such effects of tryptase may lead to altered gene expression patterns in breast cancer cells. Indeed, transcriptomic analysis revealed that tryptase caused marked effects on gene expression in breast cancer cells. Notably, among the downregulated genes were several genes recognized as pro-tumorigenic factors for breast cancer, as exemplified by CCN1 (encoding cellular communication network factor 1), CCN2 (encoding cellular communication network factor 2/connective tissue growth factor) and EDN1 (encoding endothelin 1) [41, 42]. Hence, a potential scenario behind the downregulatory effect of tryptase on breast cancer proliferation could be that tryptase, possibly through effects on epigenetic histone modification, causes downregulated expression of such genes. However, further experimentation is warranted to evaluate whether the effects of tryptase on the breast cancer cells is explained by this type of mechanism.

To provide further mechanistic insight into the effects of tryptase on breast cancer cells, we performed ATAC-seq analysis to assess whether tryptase can affect the accessibility of chromatin, thereby potentially affecting gene expression. Indeed, we noted that treatment of breast cancer cells with tryptase affected the chromatin accessibility in several regions, including reduced chromatin accessibility in regions containing genes whose transcription were suppressed by tryptase (e.g., CCN1, CCN2). Hence, we may propose that tryptase affects chromatin accessibility by proteolytic action, in turn resulting in altered expression of genes located in tryptase-susceptible regions of the chromatin.

It was noted that genes downregulated by tryptase were consistently associated with the H3K27ac mark. H3K27ac is known to be a marker for active enhancers [45] and erasure of this mark would thus result in chromatin condensation and inhibited gene transcription. Intriguingly, our comparison of the levels of this mark in control- vs. tryptase-treated breast cancer cells indicated that tryptase caused a reduction in the levels of this particular histone mark. Collectively, our findings are thus compatible with a scenario in which tryptase truncates H3 and thereby erases H3K27ac (and possibly other histone marks), leading to decreased chromatin accessibility in the corresponding genomic regions. In turn, this would result in reduced expression of the corresponding genes.

In addition to causing closure of chromatin at such distinct genomic regions, our findings reveal that tryptase treatment predominantly causes an increased chromatin accessibility. Hence, it would be expected that this may result in increased expression of multiple genes. However, the transcriptomic analyses indicated only modest induction of a limited number of genes. Although we cannot with certainty explain this seeming discrepancy, it should be emphasized that chromatin decondensation does not necessarily lead to increased gene expression, as gene expression is also dependent on many other factors, such as availability of appropriate transcription factors. Nevertheless, the TEM analysis indeed supports the notion that tryptase causes extensive decondensation of the chromatin, possibly associated with conversion of condensed chromatin into a beads-on-a-string appearance where the individual nucleosomes become separated [52]. Most likely, the cleavage of nucleosomal core histones by tryptase contributes to this, but we cannot exclude that this effect is dependent on modification of additional proteolytic targets. However, future investigations are warranted to provide a more comprehensive insight into the degradome for tryptase within breast cancer cells. Further, more extended analyses are required to clarify the mechanism of tryptase uptake into breast cancer cells, as well as to clarify how tryptase enters the cancer cells nuclei.

Altogether, the present study reveals an anti-tumorigenic impact of MC tryptase on breast cancer cells. Further, the study introduces a mechanism of action in which tryptase enters the nuclei of target tumour cells where it causes proteolytic modification of nuclear proteins, in turn leading to effects on gene expression that may account for the anti-proliferative effect of tryptase on the cells. It is clear that these findings may have an impact on our understanding of how MCs influences breast cancer. Further, the present findings may potentially be adapted for future therapeutic purposes, in which tryptase may be exploited as an anti-cancer agent in breast cancer.

## Material and methods

### Antibodies

Anti-mast cell tryptase [AA1] (Abcam, ab2378), anti-Mcpt6 immune serum [53], anti-Ki67 [SolA15] (eBioscience, #14-5689-82), Anti-GAPDH [6C5] (Santa Cruz Biotechnology, sc-32233), Anti-histone H2A (Abcam, ab18255), Anti-histone H2B (Abcam, ab1790), Anti-histone H3 (Abcam, ab1791), Anti-histone H4 (Abcam, ab10158), Anti-monomethyl-histone H3 (Lys4) [D1A9] (Cell Signaling, mAb#5326), Anti-dimethyl-histone H3 (Lys9) (Merck, #07-441), Anti-trimethyl-histone H3 (Lys9) (Abcam, ab8898), Anti-trimethyl-histone H3 (Lys27) (Abcam, ab6002), Anti-acetyl-histone H3 (Lys9) (Abcam, ab10812), Anti-acetyl-histone H3 (Lys14) (Cell Signaling, mAb#4318), Anti-histone H3 pan-acetyl (Active Motif, #39139), Anti-acetyl-histone H3 (Lys27) (Active Motif, #39133), Anti-CYR61/CCN1 [D4H5D] (Cell Signaling, mAb#14479), Anti-CTGF/CCN2 [D8Z8U] (Cell Signaling, mAb#86641), Anti-CMA1/chymase (Atlas Antibodies, HPA052634).

### Hs578T cells

The Hs578T triple-negative human breast cancer cell line (ATCC) was cultured in Dulbecco’s modified Eagle’s medium (Gibco), supplemented with 10% heat-inactivated foetal bovine serum (Invitrogen), 50 μg/ml streptomycin sulphate (Sigma-Aldrich), 60 μg/ml penicillin G (Sigma-Aldrich), 2 mM L-glutamine (Sigma-Aldrich). Cells were kept at 37 °C in 5% CO_2_ and medium was replaced when cells achieved 90-100% confluency.

### BT-549 cells

The BT-549 triple-negative human breast cancer cell line (ATCC) was cultured in RPMI-1640 (Gibco), supplemented with 10% heat-inactivated foetal bovine serum (Invitrogen), 50 μg/ml streptomycin sulphate (Sigma-Aldrich), 60 μg/ml penicillin G (Sigma-Aldrich), 2 mM L-glutamine (Sigma-Aldrich) and 10 μg/mL recombinant human insulin (Sigma-Aldrich). Cells were kept at 37 °C in 5% CO_2_ and medium replaced when cells achieved 90–100% confluency.

### BT-20 cells

The BT-549 triple-negative human breast cancer cell line (ATCC) was cultured in cultured in Dulbecco’s modified Eagle’s medium (Gibco), supplemented with 10% heat-inactivated foetal bovine serum (Invitrogen), 50 μg/ml streptomycin sulphate (Sigma-Aldrich), 60 μg/ml penicillin G (Sigma-Aldrich), 2 mM L-glutamine (Sigma-Aldrich). Cells were kept at 37 °C in 5% CO_2_ and medium replaced when cells achieved 90–100% confluency.

### MCF10A cells

The MCF10A immortalized non-tumorigenic human breast epithelial cell line (ATCC) was cultured in DMEM/F12 (Thermo Fisher), supplemented with 5% heat-inactivated foetal bovine serum (Invitrogen), 50 μg/ml streptomycin sulphate (Sigma-Aldrich), 60 μg/ml penicillin G (Sigma-Aldrich), 2 mM L-glutamine (Sigma-Aldrich), 20 ng/mL recombinant human EGF (Sigma-Aldrich), 100 ng/mL cholera toxin (Sigma- Aldrich), 0.5 μg/mL hydrocortisone (Sigma-Aldrich), 10 μg/mL recombinant human insulin (Sigma-Aldrich). Cells were kept at 37 °C in 5% CO_2_ and medium replaced when cells achieved 90–100% confluency.

### Cell viability

Cells were harvested, washed once with PBS, and resuspended in Annexin binding buffer (BD Pharmingen). They were then stained with Annexin V-FITC and Draq7, followed by analysis using a BD Accuri C6 Plus flow cytometer (BD). Viability was also assessed using PrestoBlue (Invitrogen). Cells were harvested, resuspended in culture medium, and seeded in triplicates into a 96-well plate. A 10X concentrated PrestoBlue solution was added, and the cells were incubated for 1 h at 37 °C in 5% CO₂. Fluorescence was then measured with a TECAN Infinite M200 plate reader at an excitation wavelength of 560 nm and an emission wavelength of 590 nm.

### Transmission electron microscopy (TEM)

Samples were initially fixed in a solution of 2.5% glutaraldehyde (Ted Pella) and 1% paraformaldehyde (Merck) in PIPES buffer at pH 7.4 and stored at 4 °C until processing. They were then rinsed in 0.1 M PBS for 10 minutes and incubated for 1 h in 1% osmium tetroxide (TAAB; Berks, UK) in 0.1 M PBS. After another rinse in 0.1 M PBS, samples underwent dehydration through a graded ethanol series (50%, 70%, 95%, and 99.9%), with each step lasting 10 minutes, followed by a 5-minute treatment in propylene oxide (TAAB). Next, samples were placed in a 1:1 mixture of Epon Resin (Ted Pella) and propylene oxide for 1 h, transferred to 100% resin, and left overnight. Finally, samples were embedded in fresh Epon resin within capsules, left for 1–2 h, and polymerized at 60 °C for 48 h. Ultrathin sections (60–70 nm) were then cut using an EM UC7 Ultramicrotome (Leica), mounted on grids, and contrasted with 5% uranyl acetate and Reynold’s lead citrate. Visualization was performed using a Tecnai™ G2 Spirit BioTwin transmission electron microscope (Thermo Fisher/FEI) operating at 80 kV, with imaging captured by an ORIUS SC200 CCD camera and Gatan Digital Micrograph software (both from Gatan Inc.)

### EdU labelling

Cells (0.1 × 10⁶ cells/ml) were seeded into wells of a 24-well plate. Cells were left untreated or treated with 50 nM human recombinant beta-tryptase (Promega) and incubated at 37 °C for 48 h. Two hours prior to cell harvesting, 10 µM EdU was added to the cultures. After incubation, cells were washed in PBS, trypsinized and harvested by centrifugation (400 x g, 5 min) and stained using the Click-iT^TM^ Plus EdU Alexa 647 Flow Cytometry Kit (ThermoFisher). Samples were then analysed using a BD Accuri C6 Plus flow cytometer (BD), with data collected from 10,000 events per sample and analysed using FlowJo software (BD Biosciences).

### Tryptase labelling

Recombinant human tryptase was labelled using the Alexa Fluor^TM^ 488 protein labelling kit (Invitrogen), according to manufacturer’s instructions.

### Time-lapse microscopy

Hs578T breast cancer cells (0.1 × 10⁶ cells/ml) were seeded into a 24-well plate and left to attach for approximately 2 h at 37 °C and 5% CO_2_ into an incubation chamber at a time-lapse Eclipse Ti microscope equipped with NIS-Elements software (Nikon). Cells were either left untreated or treated with 50 nM Alexa-488-labelled tryptase. Plates were then incubated for 1 h. Next, combined bright-field and fluorescent images were acquired every 2 min for a period of 30 min.

### Laser-scanning microscopy

Aliquots of 100 µl from cell suspensions (10^5^ cells/ml) were left untreated or treated with 50 or 100 nM recombinant human tryptase in 8-chamber tissue culture glass slides (Corning). After treatment, supernatants were removed, rinsed in PBS, and fixed with 4% paraformaldehyde in PBS for 15 minutes. To permeabilize the cells, 100 μl of 0.1% Triton-X100 in PBS was added to each slide, followed by incubation for 10 min at room temperature. Slides were then washed twice in PBS/1% BSA and blocked with 10% goat serum in PBS for 30 minutes at room temperature. Next, 100 μl of mouse anti-human mast cell tryptase (AA1; Abcam) diluted 1:100 in TBS/1% BSA or isotype control at the same concentration was added and incubated overnight at 4 °C. Following incubation, the slides were washed three times with TBS. Goat anti-mouse Alexa-488-conjugated antibody (diluted 1:1000 in PBS/1% BSA) was then added, followed by incubation for 1 h at room temperature in dark. Slides were washed three times with PBS/1% BSA and stained with ActinRed-555 (Invitrogen) and Hoechst 33342 (NucBlueTM, Invitrogen) for 10 minutes, followed by three washes with PBS. Slides were mounted using SlowFade® Gold Antifade Mounting Medium (Life Technologies), and analysed using a LSM 710 laser-scanning microscope equipped with ZEN 2009 software (Carl Zeiss). Z-stack sections were acquired and used for 3D image assembly and analysis with Imaris software (Oxford Instruments).

### Subcellular fractionation

Hs578T cells (1 × 10⁶) were diluted to a final concentration of 0.2 × 10⁶ cells/ml in DMEM (Gibco), supplemented with 1% heat-inactivated foetal bovine serum (Invitrogen), 50 µg/ml streptomycin sulphate (Sigma-Aldrich), 60 µg/ml penicillin G (Sigma-Aldrich), and 2 mM L-glutamine (Sigma-Aldrich). Cells were seeded into a TC-60 dish (Corning) and incubated overnight at 37 °C in a humidified atmosphere containing 5% CO₂. The following day, cells were either left untreated (control) or treated with 50 nM recombinant human tryptase for 48 hours. After treatment, the culture medium was removed, and cells were washed with PBS, trypsinized, and harvested by centrifugation at 400 × g for 5 min. Cell pellets were washed twice with PBS and processed for subcellular fractionation using the Subcellular Protein Fractionation Kit for Cultured Cells (Thermo Scientific), following the manufacturer’s instructions. The resulting fractions were collected into pre-chilled tubes and stored at −18 °C until further use.

### Tryptase ELISA

Subcellular fractions from untreated and 50 nM tryptase-treated Hs578T cells were analysed using the Human Tryptase beta-2/TPSB2 ELISA Kit (Thermo Scientific), following the manufacturer’s protocol. The assay was performed in two independent experiments, each conducted in duplicate. Data from both experiments were pooled for analysis.

### PyMT tumour sections

Randomisation or blinding was not used. Mice were on FVB/n genetic background (females; 14 weeks). Paraffin-embedded PyMT murine breast cancer tumour sections [54] were deparaffinized and hydrated. The sections were then fixed with 4% paraformaldehyde in TBS for 15 minutes, washed twice in PBS, permeabilized with 0.1% Triton-X100 in TBS for 10 minutes at room temperature. The slides were then washed twice in TBS/1% BSA and blocked with 10% goat serum in TBS for 30 minutes at room temperature. Next, rabbit anti-mouse tryptase (Mcpt6) immune serum diluted 1:500 in TBS/1% BSA or pre-immune serum control at the same concentration was added and incubated overnight at 4 °C. Following incubation, the slides were washed three times with TBS/1% BSA. Goat anti-rabbit Alexa-488-conjugated antibody in TBS/1% BSA was then added and incubated for 1 h at room temperature in the dark. Next, Rabbit anti-mouse Alexa-647-Ki67 (Novus biologicals) diluted 1:500 TBS/1% BSA or isotype control at the same concentration was added and incubated overnight at 4 °C. Following incubation, the slides were washed three times with TBS/1% BSA and stained with Hoechst 33342 (NucBlue^TM^; Invitrogen) for 10 minutes, followed by three washes with TBS. The slides were mounted using SlowFade® Gold Antifade Mounting Medium (Life Technologies). Samples were analysed using a LSM 710 laser-scanning microscope equipped with ZEN 2009 software (Carl Zeiss). Z-stack sections were acquired and used for 3D image assembly and analysis with Imaris software (Oxford Instruments). Data are representative of 7 analysed tissue sections.

### Human breast cancer tumour sections

Tissue sections from triple negative breast cancer and adjacent healthy tissue were obtained (Uppsala Biobank; ethical approval: Etikprövningsmyndigheten; no 2020-00238). Paraffin-embedded tissue sections were deparaffinized and hydrated. Sections were then fixed with 4% paraformaldehyde in PBS for 15 minutes, washed twice in PBS, permeabilized with 0.1% Triton-X100 in TBS for 10 min at room temperature. Slides were then washed twice in TBS/1% BSA and blocked with 10% goat serum in TBS for 30 minutes at room temperature. Next, mouse anti-human mast cell tryptase (AA1; Abcam; diluted 1:100 in TBS/1% BSA) or isotype control at the same concentration was added, followed by incubation overnight at 4 °C. Next, the slides were washed three times with TBS/1% BSA. Goat anti-mouse Alexa-488-conjugated antibody (diluted 1:1000 in TBS/1% BSA) was then added and incubated for 1 h at room temperature in the dark. Next, rabbit anti-human Alexa-647-Ki67 (Novus biologicals) diluted 1:500 TBS/1% BSA or isotype control at the same concentration was added and incubated overnight at 4 °C. The slides were then washed three times with TBS/1% BSA and stained with Hoechst 33342 (NucBlue^TM^, Invitrogen) for 10 min, followed by three washes with TBS. Slides were mounted using SlowFade® Gold Antifade Mounting Medium (Life Technologies). Samples were analysed using a LSM 710 laser-scanning microscope equipped with ZEN 2009 software (Carl Zeiss). Z-stack sections were acquired and used for 3D image assembly and analysis with Imaris software (Oxford Instruments). Data are representative of 15 analysed tissue sections.

### Fluorescence microscopy

Frozen tissue sections from a triple-negative human breast cancer tumour were fixed and permeabilized with ice-cold methanol for 15 minutes. Slides were then washed twice with PBS containing 1% BSA (PBS/1% BSA) and blocked with 5% goat serum in PBS for 30 minutes at room temperature. Subsequently, slides were incubated overnight at 4 °C with rabbit anti-human mast cell chymase 1 antibody (CMA1; Atlas Antibodies) diluted 1:500 in PBS/1% BSA. Negative controls were prepared by incubating sections with PBS/1% BSA only. After five washes in PBS/1% BSA, slides were incubated for 1 h at room temperature with goat anti-rabbit Alexa Fluor 488 antibody diluted 1:1000 in PBS/1% BSA. Following three additional washes, slides were incubated overnight at 4 °C with mouse anti-human mast cell tryptase antibody (AA1; Abcam) diluted 1:100 in PBS/1% BSA. Corresponding negative controls were prepared in parallel. Slides were then washed five times in PBS/1% BSA and incubated for 1 h at room temperature with goat anti-mouse Alexa Fluor 555 antibody diluted 1:1000 in PBS/1% BSA. After three final washes in PBS/1% BSA, nuclei were stained with Hoechst 33342 (NucBlue™, Invitrogen) for 10 min. Slides were washed again, dried at room temperature, and mounted using SlowFade® Gold Antifade Mountant (Life Technologies). Samples were imaged using a Nikon 90i fluorescence microscope equipped with NIS-Elements software (Nikon).

### Western blot analysis

Equal numbers of cells were collected and solubilized in Laemmli buffer (Bio-Rad). The samples were then subjected to SDS-PAGE using Bio-Rad 4–20% Mini-Protean TGX stain-free gels, followed by transfer to a 0.2 µm nitrocellulose membrane (Trans-Blot Turbo Transfer Pack, Bio-Rad) using the Bio-Rad Trans-Blot Turbo transfer system. Membranes were blocked with EveryBlot blocking buffer (Bio-Rad), incubated with primary antibody overnight at 4 °C, and then with a StarBright B700 secondary antibody (Bio-Rad; diluted 1:5000 in TBS) for 1 h at room temperature. The membranes were scanned using a ChemiDoc^TM^ MP imaging system equipped with Image Lab^TM^ Touch Software (Bio-Rad).

### Ampliseq transcriptome analysis

Hs578T (1 × 105 cells) were incubated in quadruplicates in wells of a 12-well plate for 6 or 24 h, with or without 50 nM tryptase. Cells were harvested and total RNA was isolated using the NucleoSpin RNA kit (Macherey-Nagel) according to the manufacturer’s instructions. The samples were analysed at NGI Sweden (Uppsala). cDNA libraries were generated and amplified using the Ion AmpliSeqTM Transcriptome Human Gene Expression Kit (Life Technologies), following the protocol provided by the manufacturer, and sequencing was performed on an Ion S5TM XL Sequencer (Thermo Fisher Scientific). Normalized expression values generated by Torrent SuiteTM Software (version 5.10.1) were used for downstream differential expression analyses using the edgeR package (Robinson, McCarthy, and Smyth 2010) implemented in R (version 3.6.0) (The R Foundation n.d.). The full data set is available: GEO Submission (GSE307724); NCBI tracking system #25363733.

### ATAC-seq analysis

Hs578T cells (0.5 × 10^5^ cells) were seeded into wells of a 24-well plate. Triplicates of cells were left untreated or treated with 50 nM recombinant human skin tryptase (Promega) at 37 °C for 48 h. Cells were then harvested by centrifugation at 500 x g for 10 min at 4 °C and cell pellets were resuspended in 50 μl of cold lysis buffer (10 mM Tris HCl, pH 7.4, 10 mM NaCl, 3 mM MgCl_2_, 0.1% NP-40, 0.1% Tween-20 and 0.01% digitonin) and incubated on ice for 3 min. Pellets were resuspended with 1 ml wash buffer (10 mM Tris HCl, pH 7.4, 10 mM NaCl, 3 mM MgCl_2_, 0.1% Tween-20) and cell nuclei were pelleted at 500 x g for 10 min at 4 °C. Supernatants were removed and pellets were resuspended in 50 μl of tagmentation master mix (TD buffer, 0.5% tween 20, 0.5% digitonin and TDE1 enzyme). Samples were incubated for 30 min at 37 °C in a thermomixer 5436 (Eppendorf) with a mixing speed of 1000 rpm, followed by adding 250 μl of ChIP DNA binding buffer to the tagmented DNA. The mixture was transferred to a Zymo-Spin^TM^ IC-XL column in a collection tube, centrifugated for 30 sec at 13,000 x g and the flow-through was discarded. The column was washed twice with 200 μl DNA washing buffer (1 min 13,000 x g) and samples were eluted with 15 μl DNA elution buffer directly to the column matrix (30 sec; 13,000 x g). The eluted DNA samples were transferred to a TwinTec 96-well plate. Samples were sequenced on NextSeq2000 (NextSeq 1000/2000 Control Software 1.5.0.42699/RTA 3.10.30) with a 51nt(Read1)-8nt(Index1)-8nt(Index2)-51nt(Read2) setup using ‘P2’ flowcell. Bcl to FastQ conversion was performed using bcl2fastq_v2.20.0.422 from the CASAVA software suite. The quality scale used is Sanger / phred33 / Illumina 1.8 + .

The ATAC-seq analysis was performed using the Nextflow pipeline NF-core/atacseq version 2.1.2 (https://nf-co.re/atacseq) [55]. Briefly, sequence adapters are trimmed by Trim Galore, and aligned by BWA. Picard marked duplicates and merged alignments from multiple libraries to one sample, mitochondrial DNA, duplicates, unmapped regions and other unwanted sequences are filtered by SAMtools, Pysam and BAMTools. Normalized bigWig files were generated by BEDTools, and bedGraphToBigWig. Genome-wide enrichment was done by deepTools and peaks were called by MACS2. Differential accessibility analysis was performed by DESeq2 and Homer was used to annotate gene features. The complete dataset is available at the Sequence Read Archive (NCBI; accession number: PRJNA1180776).

### Real-Time qPCR

RNA was extracted using the NucleoSpin RNA kit (Macherey-Nagel) according to the protocol provided by the manufacturer. RNA quality and concentration were determined using a Nanodrop spectrophotometer (Thermo Fisher Scientific). Subsequently, cDNA was prepared by using the iScript cDNA Synthesis kit (Bio-Rad), following the instructions provided by the manufacturer. cDNA was synthesized using the SimpliAmp thermocycler (Life Technologies). RT-qPCRs analyses were performed with technical quadruplicates, using the iTaq universal SYBR green supermix kit (Bio-Rad). Samples were analysed using 384-well plates, with a CFX384 real-time system (Bio-Rad). Samples were analysed using the CFX Maestro software (Bio-Rad). The expression of each gene was given relative to GAPDH, with the use of the formula 2(-ΔCq). The used primers were: CCN1-Forward: 5′- ACCGCTCTGAAGGGGATCT-3′, CCN1-Reverse: 5′ ACTGATGTTTACAGTTGGGCTG-3′, CCN2-Forward: 5′ -AAAAGTGCATCCGTACTCCCA-3′ and CCN2-Reverse: 5′- CCGTCGGTACATACTCCACAG-3 ´.

### Statistical analyses

Statistical analyses were performed by using the GraphPad Prism 10 software.

## Supplementary information

Suppl. Fig. 1

Suppl. Fig. 2

Uncropped gels

Suppl Video 1

Suppl Video 2

## Data Availability

All data generated and analysed during this study are included in this article. Further details or raw data are available from the corresponding authors upon reasonable request. The full Ampliseq data set is available: GEO Submission (GSE307724); NCBI tracking system #25363733. The complete ATAC-seq dataset is available: GEO Submission (GSE308338).
