# Supplementary material for: Mast cell tryptase induces nuclear remodelling and reduced growth in breast cancer cells
Source: Cell Death Discov. 2025 Oct 27;11:485. doi: 10.1038/s41420-025-02813-1 (PMC12559418; doi:10.1038/s41420-025-02813-1)
Supplement: Supplementary file 1 — Suppl. Fig. 1 [file 41420_2025_2813_MOESM1_ESM.pdf]

Human breast cancer tumor tissue

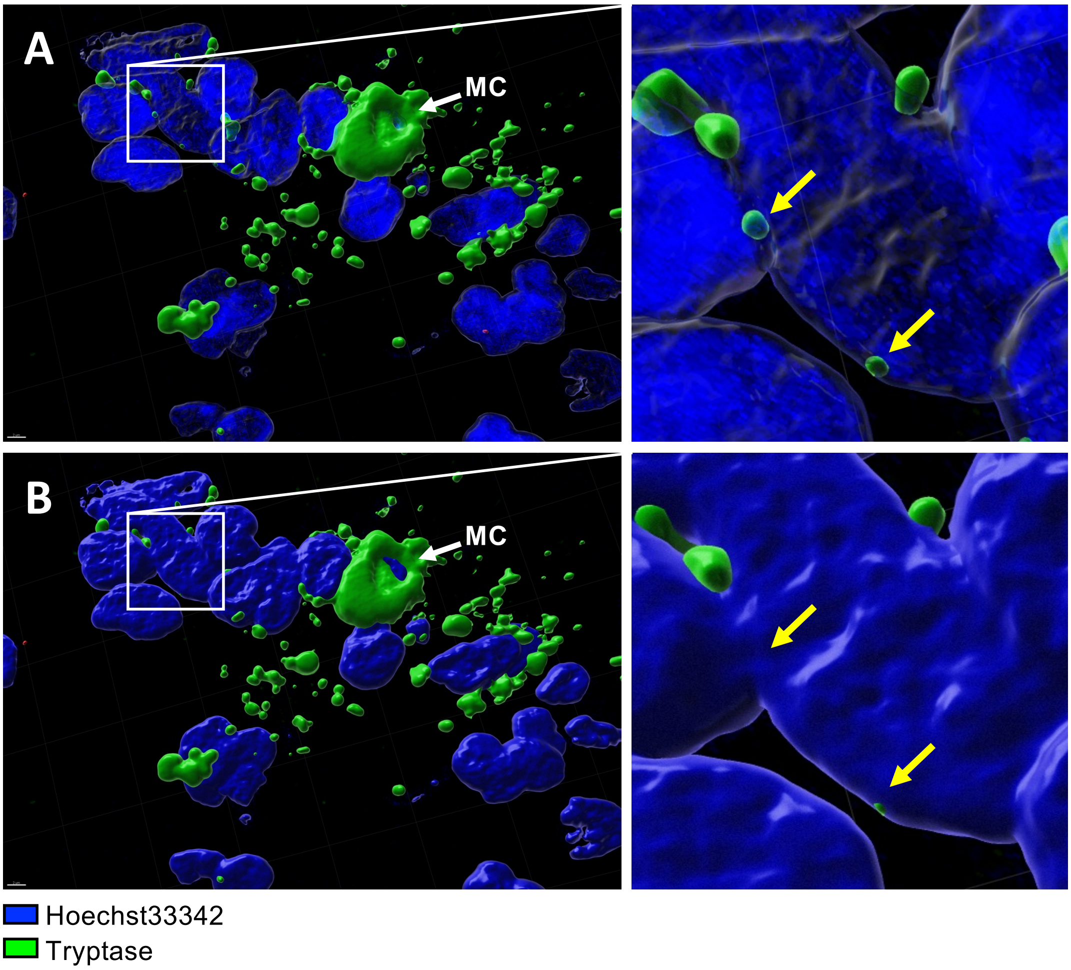

Suppl. Fig. 1. Nuclear localization of tryptase in human breast cancer tumour cells. Confocal Z-stack images stained with Hoechst 33342 (blue), tryptase (green) were used to create 3D reconstructions of human triple-negative breast cancer tumours. White arrows indicate mast cell (MC) presence. (A) Section of a human breast cancer tumour highlighting translucent nuclear 3D structures with nuclear tryptase localization (yellow arrows). (B) Section of a human breast cancer tumour featuring solid block nuclear 3D structures where tryptase staining was suppressed (yellow arrows), indicating the nuclear localization.
