# Supplementary material for: Mast cell tryptase induces nuclear remodelling and reduced growth in breast cancer cells
Source: Cell Death Discov. 2025 Oct 27;11:485. doi: 10.1038/s41420-025-02813-1 (PMC12559418; doi:10.1038/s41420-025-02813-1)
Supplement: Supplementary file 2 — Suppl. Fig. 2 [file 41420_2025_2813_MOESM2_ESM.pdf]

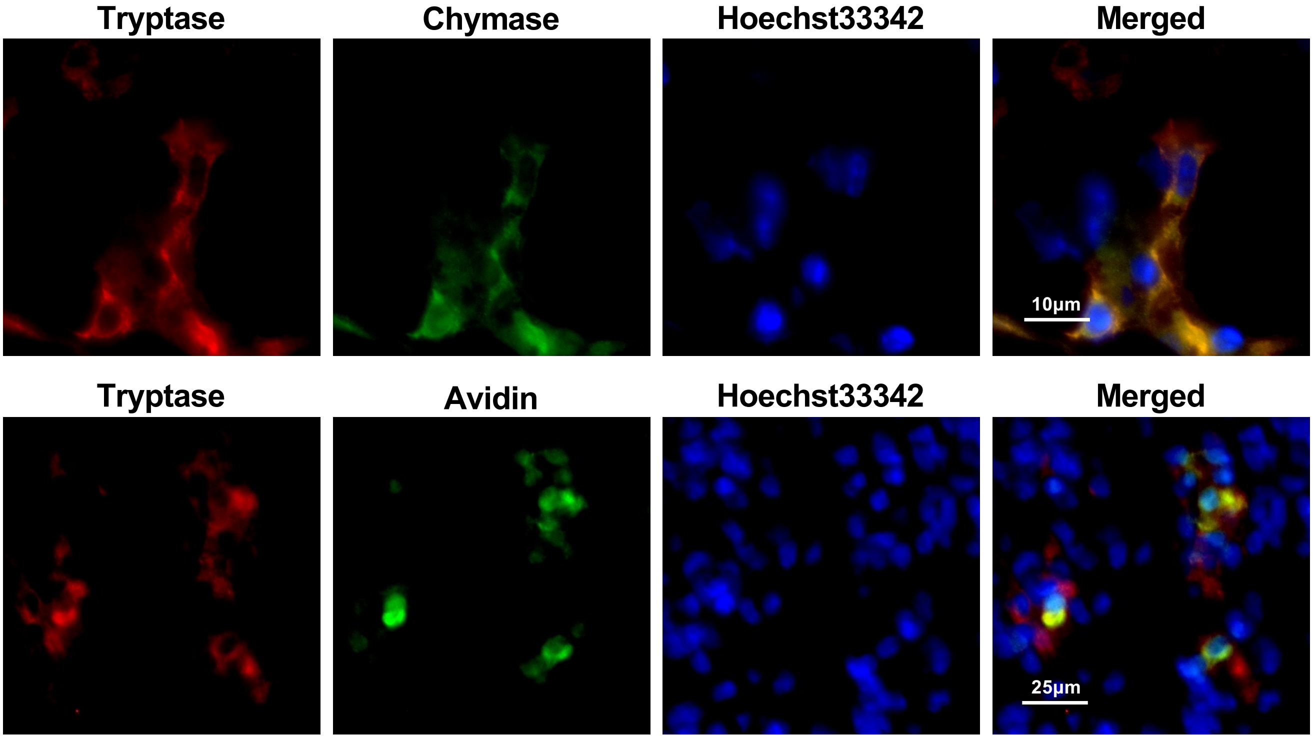

Suppl. Fig. 2. Immunofluorescent staining showing colocalization of tryptase with chymase and avidin in human triple-negative breast cancer tissue sections. Sections were co-stained for tryptase and chymase (top panels) or tryptase and avidin (bottom panels). Nuclei were counterstained with Hoechest 33342. Scale bar: 20 µm.
