# Supplementary figures and images for: Mast cell tryptase induces nuclear remodelling and reduced growth in breast cancer cells
Source: Cell Death Discov. 2025 Oct 27;11:485. doi: 10.1038/s41420-025-02813-1 (PMC12559418; doi:10.1038/s41420-025-02813-1)

**Fig.6 (A)**

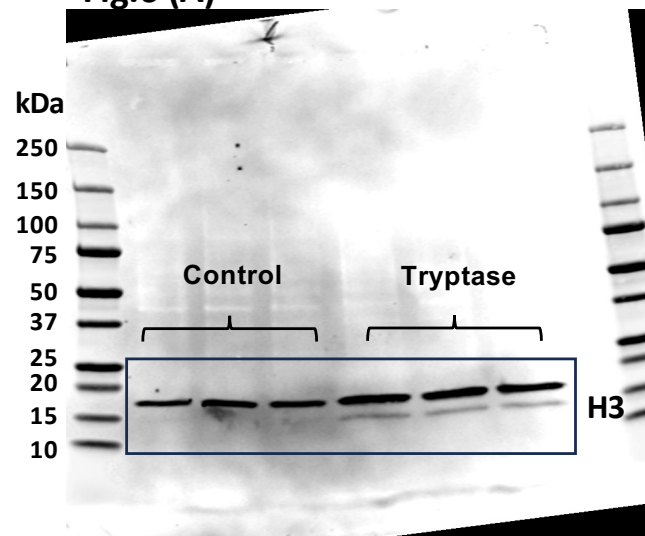

**Fig.6 (B)**

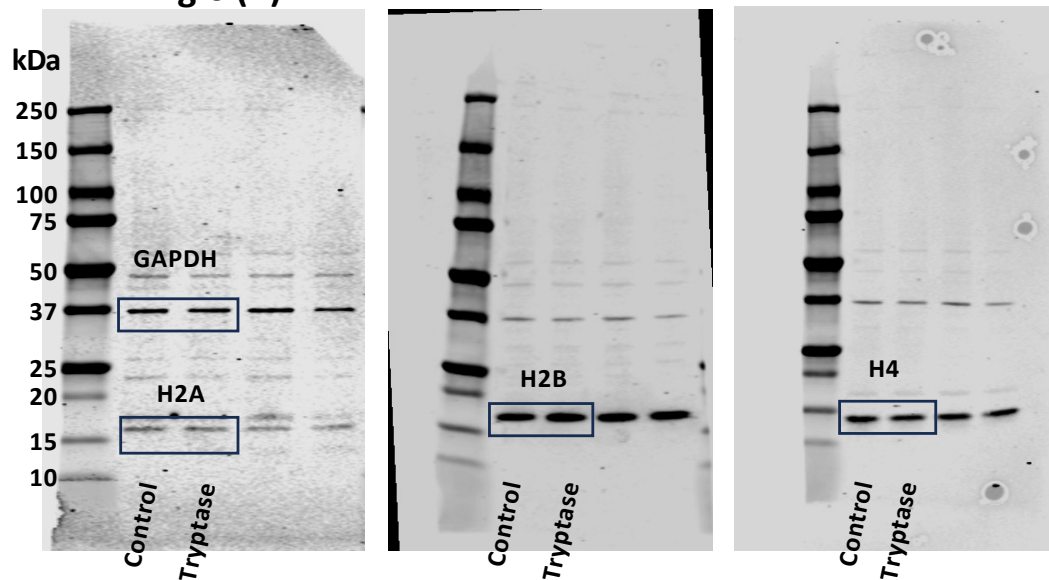

**Fig.6 (C)**

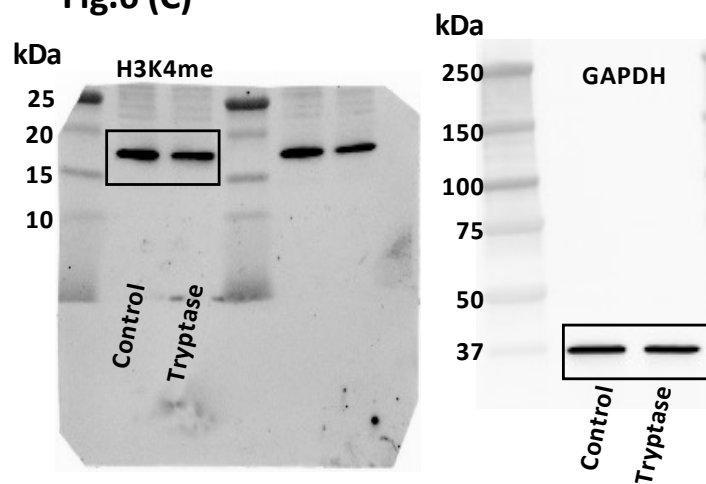

**Fig.6 (D)**

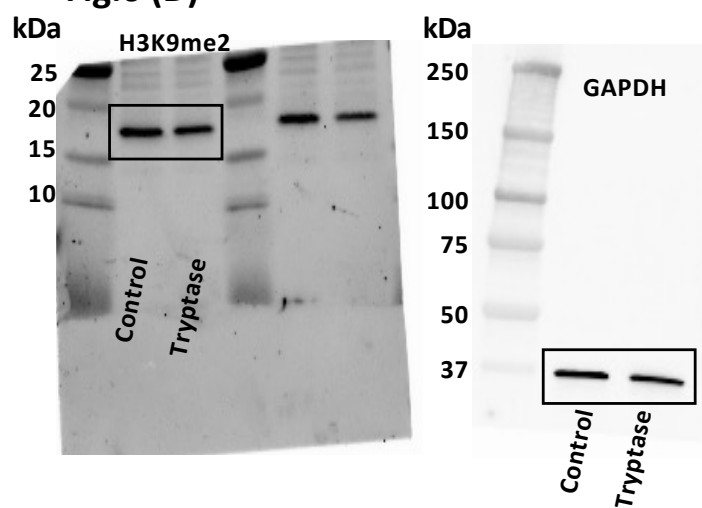

**Fig.6 (E)**

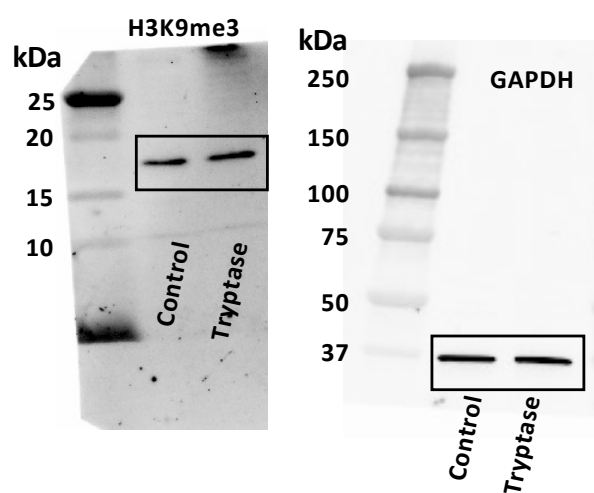

**Fig.6 (F)**

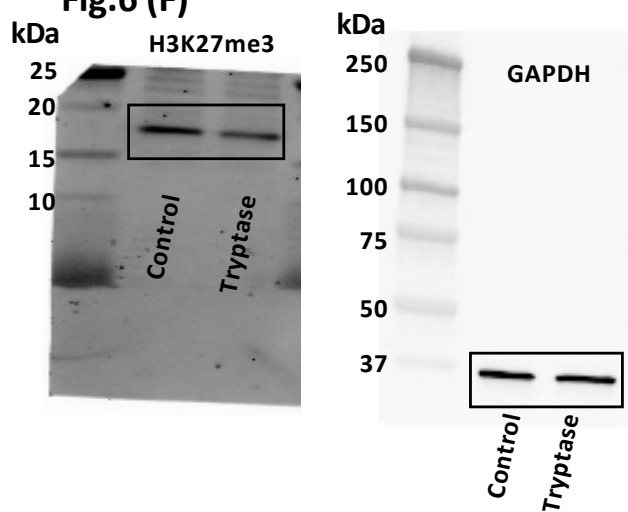

**Fig.6 (G)**

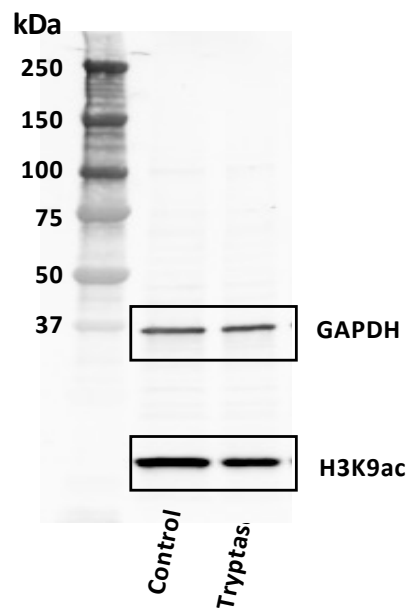

**Fig.6 (H)**

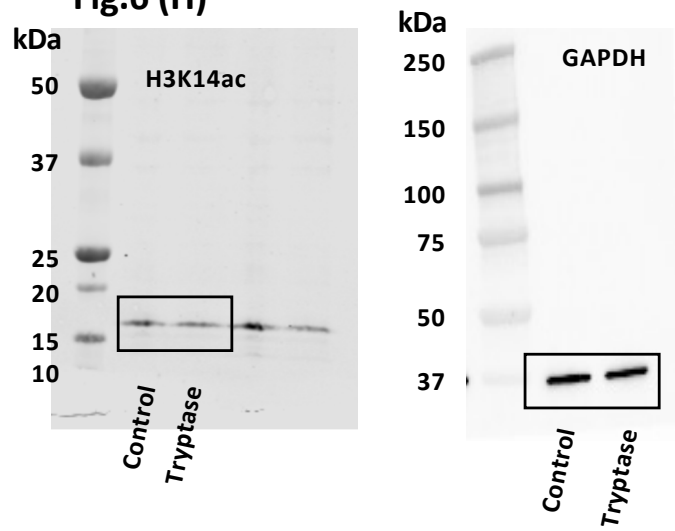

**Fig.6 (I)**

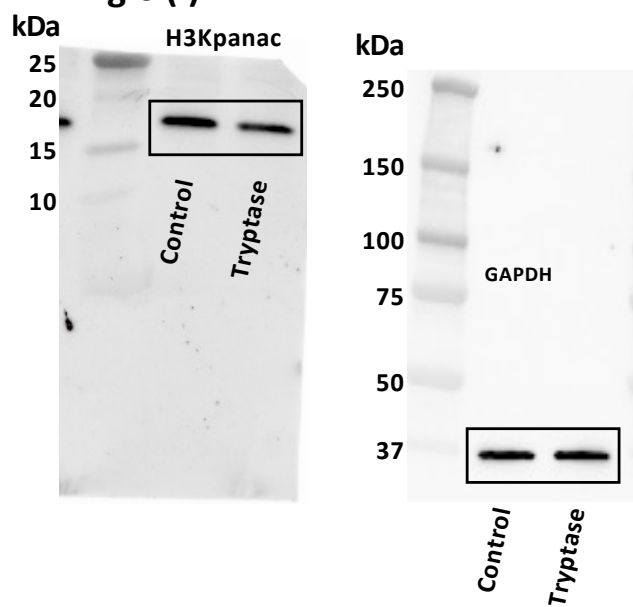

**Fig.7 (D)**

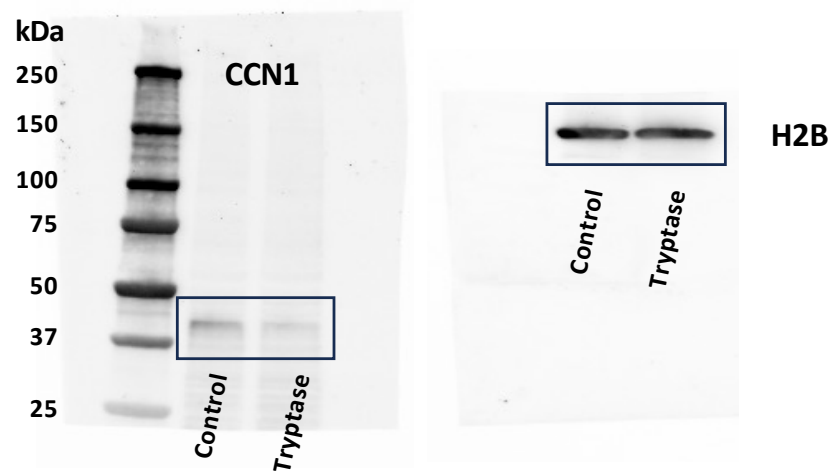

**Fig.7 (D)**

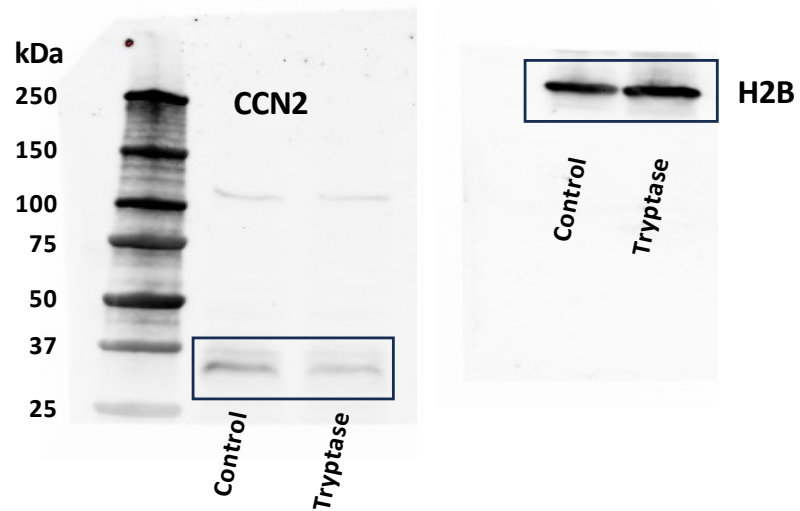

**Fig.8 (F)**

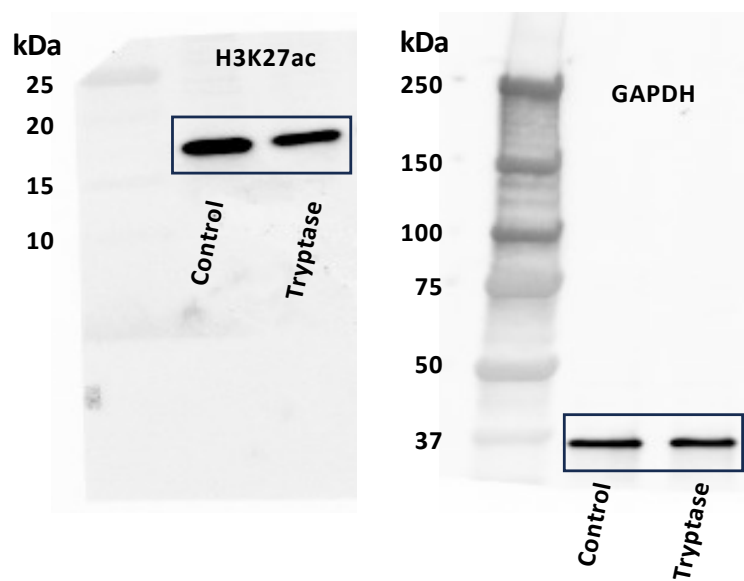

Supplement: Supplementary file 3 — Uncropped gels [file 41420_2025_2813_MOESM3_ESM.pdf]
